# Keras R-CNN: library for cell detection in biological images using deep neural networks

**DOI:** 10.1186/s12859-020-03635-x

**Published:** 2020-07-11

**Authors:** Jane Hung, Allen Goodman, Deepali Ravel, Stefanie C. P. Lopes, Gabriel W. Rangel, Odailton A. Nery, Benoit Malleret, Francois Nosten, Marcus V. G. Lacerda, Marcelo U. Ferreira, Laurent Rénia, Manoj T. Duraisingh, Fabio T. M. Costa, Matthias Marti, Anne E. Carpenter

**Affiliations:** 1 Department of Chemical Engineering, Massachusetts Institute of Technology, Cambridge, MA USA; 2 The Broad Institute, Cambridge, MA USA; 3 Harvard T.H.Chan School of Public Health, Boston, MA USA; 4 Instituto Leônidas e Maria Deane, Fundação Oswaldo Cruz (FIOCRUZ), Manaus, Amazonas Brazil; 5 Fundação de Medicina Tropical Dr. Heitor Vieira Dourado, Gerência de Malária, Manaus, Amazonas Brazil; 6 Universidade de São Paulo, São Paulo, Brazil; 7 Department of Microbiology & Immunology, Yong Loo Lin School of Medicine, National University of Singapore, Singapore, 119077 Singapore; 8 Singapore Immunology Network (SIgN), Agency for Science Research & Technology, Singapore, 138632 Singapore; 9 Shoklo Malaria Research Unit, Mahidol-Oxford Tropical Medicine Research Unit, Faculty of Tropical Medicine, Mahidol University, Mae Sot, Thailand; 10 Centre for Tropical Medicine and Global Health, Nuffield, Oxford, UK; 11 Department of Genetics, Evolution, Microbiology and Immunology, University of Campinas, Campinas, SP Brazil; 12 Wellcome Centre for Integrative Parasitology Institute of Infection, Immunity and Inflammation, College of Medical Veterinary & Life Sciences, University of Glasgow, Glasgow, UK

**Keywords:** Deep learning, Keras, Convolutional networks, Malaria, Object detection

## Abstract

**Background:**

A common yet still manual task in basic biology research, high-throughput drug screening and digital pathology is identifying the number, location, and type of individual cells in images. Object detection methods can be useful for identifying individual cells as well as their phenotype in one step. State-of-the-art deep learning for object detection is poised to improve the accuracy and efficiency of biological image analysis.

**Results:**

We created *Keras R-CNN* to bring leading computational research to the everyday practice of bioimage analysts. *Keras R-CNN* implements deep learning object detection techniques using Keras and Tensorflow (https://github.com/broadinstitute/keras-rcnn). We demonstrate the command line tool’s simplified Application Programming Interface on two important biological problems, nucleus detection and malaria stage classification, and show its potential for identifying and classifying a large number of cells. For malaria stage classification, we compare results with expert human annotators and find comparable performance.

**Conclusions:**

*Keras R-CNN* is a Python package that performs automated cell identification for both brightfield and fluorescence images and can process large image sets. Both the package and image datasets are freely available on GitHub and the Broad Bioimage Benchmark Collection.

## Background

**Fig. 1:**
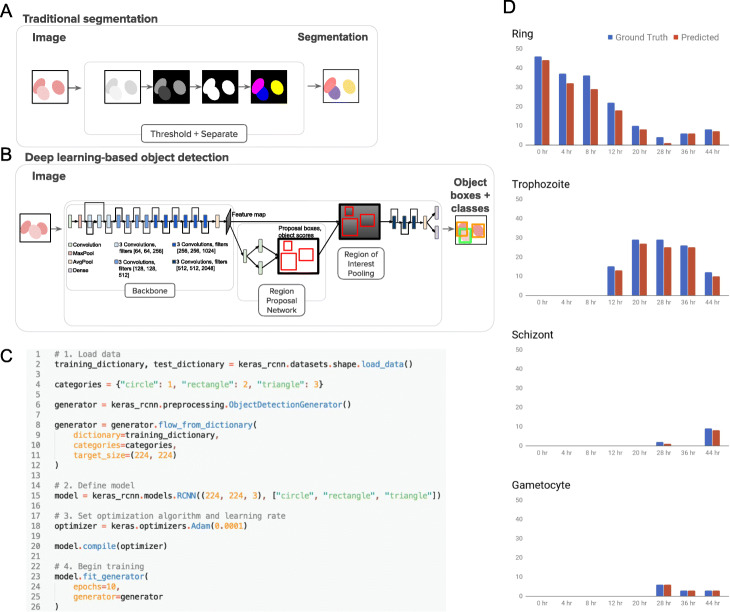
Overview of a traditional segmentation based pipeline and a deep learning based object detection pipeline. **a**. Traditional segmentation based pipelines require the selection and tuning of multiple classical image processing algorithms to produce a segmentation, where pixels associated with individual instances (e.g. nuclei, or cells) receive unique “labels”, represented here as different colors. **b**. Deep learning-based object detection pipelines require some example annotated images to be provided, and use neural networks to learn a model that can produce bounding boxes around each object, which can be overlapping. If multiple object classes are of interest (for example, multiple phenotypes), each bounding box is assigned a class. **c**. Code to train an object detection model, written using Keras R-CNN’s API. **d**. Graphs of cell counts of each infected type over time predicted on time course images. The time course set contains samples prepared at particular hours between 0 and 44 h and has been designed to synchronize the parasites’ growth and to show representation of all stages. The ground truth is based on Annotator 1, who annotated all images in the dataset including the training data

Identifying individual cells in images is often a crucial task for basic biology research, high-throughput drug screening and digital pathology. Traditional segmentation methods (Fig. 1a) identify individual pixels that belong to each distinct object through a carefully designed series of image processing steps, often involving watershed, distance transform, and intensity gradients. This approach requires algorithm selection and parameter tuning (and thus time and expertise), is computationally expensive, and often fails to sufficiently handle noisy images, illumination fluctuations, and clumped cells. In cases where phenotype classification is also needed, hundreds of classical morphological features are extracted per cell, followed by a subsequent machine learning step to classify each cell. User-friendly software exists for these steps [1–4], but it does add significant effort to a typical workflow.

Deep learning holds tremendous promise to overcome these challenges by simplifying workflows while also improving accuracy, at least in many contexts [5]. In particular, deep learning-based object detection algorithms (Fig. 1b) examine the raw pixels of images and discover which features and combinations of features best describe each phenotypic class of cells (based on examples provided by the researcher), with minimal user configuration of mathematical parameters. They can conveniently identify cells as well as their phenotype in one step. In contrast to segmentation, object detection algorithms yield a bounding box around each cell. Treating cell identification as an object detection problem rather than a pixel-level segmentation problem has several advantages, most notably that the annotation step is orders of magnitude faster. Drawing bounding boxes (in essence, marking two points in space) is much faster than the pixel level annotation that is needed for ground truth for training segmentation models, and they allow overlapping objects to be easily distinguishable. Object detection algorithms additionally do not need distinct steps to separate overlapping or touching cells and require much less storage in terms of training data and results. For cases where pixel-level segmentation is needed, object detection can be followed by post-processing steps to define precise boundaries for each object.

Here, we present an open source Keras package for cell detection called *Keras R-CNN*, as well as pre-trained deep learning models and new public datasets. *Keras R-CNN* is based on the Faster Region-based Convolutional Neural Network (*Faster R-CNN*) [6] architecture, which is currently the basis of many best-performing models for object detection. It was among the top scoring in the 2018 Data Science Bowl public competition we co-organized [7] and has already compared favorably with other deep learning architectures in performance and inference time [5, 8] (Supplementary Figure S6). *Faster R-CNN* takes an image as input and generates bounding boxes and bounding box classifications [9]. Training involves gathering appropriate data as ground truth, namely a set of images with known bounding box coordinates and annotated labels annotated. Initially, a training procedure adapts the model to a particular application by optimizing the weights in the network. Subsequently, inference or prediction involves new images running through the trained model to produce output bounding box coordinates and class probabilities. To test the accuracy of a trained model for a given application, prediction is performed on a set of images that were not used for training (and, importantly, were collected in a completely different experimental batch than those used for training [10]). The predictions are compared to the known ground truth annotations to characterize accuracy.

## Implementation

*Keras R-CNN* is distinguished from other deep learning based object detection implementations like Facebook’s Detectron [11] or Tensorflow’s Object Detection API [12] in several ways. First, *Keras R-CNN* can process an unlimited number of channels. Unlike standard consumer photos’ red, green and blue (RGB) channels, biological imaging assays often involve up to several dozen fluorescent labels in multispectral imaging. The *Keras R-CNN* schema is designed for users to easily provide their own datasets; its modular structure allows for flexibility and interoperability with Keras and the scientific Python ecosystems including NumPy, and it is portable across platforms (Windows, Mac, Linux) and devices. It also includes dataset augmentation through cropping, rescaling, and rotating; and efficient handling of large scale, densely annotated, and three (or more)-dimensional datasets.

*Keras R-CNN* can train a model in just a few lines of code as compared to the hundreds or even thousands required by other implementations (Fig. 1c). While not itself a point-and-click tool, *Keras R-CNN* could serve as the foundation for a more accessible software tool serving biologists and pathologists [13]. The use of Keras offers platform and device portability while being abstract enough for the code to be understandable and easily customizable. We also designed a human-readable schema for datasets (see Supplemental Material), which is readily understood by non-experts in computer vision so they can focus on solving biological problems.

## Results

We first demonstrated *Keras R-CNN* on a very common application: nucleus detection (see Supplementary Figure S1). We used manually annotated fluorescent images showing nuclei stained with Hoechst 33342 to highlight their DNA. The ~ 600 training images (~ 30,000 nuclei) were from the 2018 Data Science Bowl (DSB) dataset *BBBC038* and the ~ 100 testing images (~ 10,000 nuclei) were from a subset of the human U2OS cell dataset *BBBC022* [14, 15]. We recently described instance segmentation of nuclei [16]; here we instead address the problem of object detection, which yields bounding boxes and whose accuracy is assessed by a different metric.

We found that the trained model achieved a mean average precision score of 82% at an intersection-over-union (IoU) threshold of 0.5. For comparison, we also tested the traditional approach of segmentation, using a CellProfiler [17] pipeline tuned to the data, achieving a score of 81%. The resulting scores are very similar, indicating that, on this relatively straightforward image analysis task, *Keras R-CNN* can perform as well as classical image processing algorithms that have been parameter-tuned by experts.

We next tested a more complex case: detection of cells in blood smears of patients infected with the human malaria parasite *Plasmodium vivax* in various stages, which requires phenotype classification (Supplementary Figure 2A) in addition to object detection. *P. vivax* causes a significant health burden in malaria endemic regions. Manual inspection of blood smears by trained microscopists remains the gold standard of parasite detection and stage determination because of its low cost and high flexibility. However, manual inspection and counting is tedious, requires resources to develop expertise, and is prone to significant human variability as the phenotypic changes are very subtle.

We applied *Keras R-CNN* to classify different stages of *P. vivax* development, with particular focus on trophozoites vs gametocytes. We collected 1364 images (~ 80,000 cells) from samples prepared by different groups: from Brazil, from Southeast Asia, and ex-vivo cultured samples prepared as a time course [18] and made them publicly available as *BBBC041* (see Supplementary Table S1). We created these three datasets across sites to increase reproducibility and robustness across different sample sets, preventing overfitting to a single laboratory’s imaging and staining routine (Supplementary Figure 2C). We used the time course images as our holdout set, and the others as training data.

We found that a trained *Keras R-CNN* model achieved a mean average precision score of 78% at an IoU threshold of 0.5 (Supplementary Figure 1), much higher than an expert-configured CellProfiler pipeline, which yielded 61%. Classical image processing struggles with this task, which is much more challenging than nucleus detection because the malaria images are brightfield rather than the high signal-to-noise fluorescent images in the nucleus detection case.

Because *Keras R-CNN* learns from expert-provided data, it is able to handle different image modalities without hand tuning parameters.

Mean average precision only assesses the ability to locate cells; we also evaluated phenotype classification. Classifying *P. vivax* stages is notoriously difficult, and often experts disagree. We quantified the level and nature of disagreement by giving two expert annotators the same set of blood smear images; there is significant confusion (disagreement) between them as well as a substantial number of cells each expert marked as low confidence (“difficult”) (Supplementary Figure 2B). In particular, trophozoites and gametocytes are prone to misclassification.

In light of this, in addition to comparing our predictions directly with expert annotations, we evaluated whether the results match the expected pattern for the time course images. Given the *P. vivax* life cycle, the number of parasites in the ring stage should decrease over time (until the parasites seed new cells and the cycle repeats), and the number in later stages should increase over time, consistent with expert counts and our results (Fig. 1d). We used diffusion pseudotime [19] to create an unsupervised ordering of all the cells in the data set based on the features learned by the model, to see if these features can capture the known progression of the various parasite stages; previously, this sort of modeling has been used to show that deep learning features are useful in capturing other chronological progressions such as cell cycle phase (Supplementary Figure S3). The ordering of the cells matches the expected cell progression, which suggests the learned features capture important and biologically meaningful underlying cell information.

## Conclusions

In summary, we find that state-of-the-art object detection with *Keras R-CNN* can be accomplished with just ~ 20 lines of code and little computer vision expertise required. As our aim was to implement an already-proven architecture, we did not focus here on optimizing and assessing model accuracy; performance could also improve even further given more annotated examples and better hyperparameter optimization. We emphasize that annotated example cell images are required to train the system, but many biologists may prefer marking bounding boxes rather than learning how to choose, operate and tune classical segmentation algorithms. The models trained in this work are freely available although tailored to specific applications; the open-source framework makes training for other applications and datasets relatively straightforward. We have also recently added additional architectures from the literature, such as Feature Pyramid Network (FPN) [20] and Mask R-CNN [21].

*Keras R-CNN’*s popularity was unexpected for a pre-1.0 release. It presently has more than 650 stars and forks on GitHub, making it one of the most popular codebases available on the Broad Institute GitHub organization. Machine learning based computer vision algorithms have the potential to greatly improve the accuracy of image analysis for biology and decrease reliance on human labor, by reducing manual image analysis as well as time spent configuring automated algorithms. *Keras R-CNN* is therefore a useful tool for performing automated cell identification for both brightfield and fluorescence images, and can serve as a foundation for future point-and-click software.

## Supplementary information

**Additional file 1: Figure S1.** Comparison of mean average precision curves for different IoU thresholds for Keras R-CNN versus CellProfiler on the nuclei and malaria datasets. For nuclei, the mean average precision is 0.99 at a threshold of 0.5 for Keras R-CNN. For malaria, the mean average precision is 0.78 at a threshold of 0.5 for Keras R-CNN. **Figure S2.** Overview of *P. vivax* data and results. The samples contain two classes of uninfected cells (red blood cells and leukocytes) and four classes of infected cells (gametocytes, rings, trophozoites, and schizonts) and have a heavy imbalance: more than 95% of all cells are uninfected, roughly the distribution in patient blood. A. Depiction of all relevant cell types found in human blood, including two types of uninfected cells and 4 types of infected cells in the *P. vivax* life cycle. The cycle on the left shows asexual development. Gametocytes come from sexual development and lead to transmission. B. Confusion matrix comparing annotations of two experts (colors normalized so that rows sum to 1); the significant signal off-diagonal speaks to the challenge for experts to agree upon the proper stage label for each cell. Experts were asked to identify relevant cells and label them as one of the cell types or difficult. C. Example of malaria-infected blood smear results. Red boxes are ground truth; blue boxes are predictions produced by Keras R-CNN. **Table S1**. Malaria datasets. **Figure S3.** Results for *P. falciparum*. **Figure S4.** Visualization of learned features and single-cell data. Diffusion pseudotime plots made from deep learning features with accompanying ground truth class information. The first row has plots of the first two diffusion coordinates and the next row has plots of the second and third diffusion coordinates. Note: the model used to generate these plots is slightly different than the final one run in the paper. **Figure S5.** Visualization of learned features and single-cell data. t-SNE plot made from deep learning features colored by ground truth class information. Note: the model used to generate these plots is slightly different than the final one run in the paper. **Figure S6.** Inference time comparison across common object detection methods.

## Data Availability

Availability and requirements: Project name: Keras R-CNN. Project home page: https://github.com/broadinstitute/keras-rcnn Operating system: Platform independent. Programming language: Python 3. Other requirements: keras-resnet, numpy, tensorflow==1.13.1, Keras==2.2.4, scikit-image==0.15.0. License: BSD. The model weights can be found in keras_rcnn.applications. JHung2019. The datasets supporting the conclusions of this article are available in the following: Malaria data are available at https://data.broadinstitute.org/bbbc/BBBC041/. Nuclei data are available at https://data.broadinstitute.org/bbbc/BBBC022/.

## Abbreviations

API: Application programming interface
BBBC: Broad Bioimage Benchmark Collection
DNA: Deoxyribonucleic acid
DSB: Data Science Bowl
FPN: Feature Pyramid Network
IoU: Intersection over union
*P. vivax*: *Plasmodium vivax*
R-CNN: Region-based convolutional neural network
