# Supplementary material for: Keras R-CNN: library for cell detection in biological images using deep neural networks
Source: BMC Bioinformatics. 2020 Jul 11;21:300. doi: 10.1186/s12859-020-03635-x (PMC7353739; doi:10.1186/s12859-020-03635-x)
Supplement: Supplementary file 1 — Additional file 1: Figure S1. Comparison of mean average precision curves for different IoU thresholds for Keras R-CNN versus CellProfiler on the nuclei and malaria datasets. For nuclei, the mean average precision is 0.99 at a threshold of 0.5 for Keras R-CNN. For malaria, the mean average precision is 0.78 at a threshold of 0.5 for Keras R-CNN. Figure S2. Overview of P. vivax data and results. The samples contain two classes of uninfected cells (red blood cells and leukocytes) and four classes of infected cells (gametocytes, rings, trophozoites, and schizonts) and have a heavy imbalance: more than 95% of all cells are uninfected, roughly the distribution in patient blood. A. Depiction of all relevant cell types found in human blood, including two types of uninfected cells and 4 types of infected cells in the P. vivax life cycle. The cycle on the left shows asexual development. Gametocytes come from sexual development and lead to transmission. B. Confusion matrix comparing annotations of two experts (colors normalized so that rows sum to 1); the significant signal off-diagonal speaks to the challenge for experts to agree upon the proper stage label for each cell. Experts were asked to identify relevant cells and label them as one of the cell types or difficult. C. Example of malaria-infected blood smear results. Red boxes are ground truth; blue boxes are predictions produced by Keras R-CNN. Table S1. Malaria datasets. Figure S3. Results for P. falciparum. Figure S4. Visualization of learned features and single-cell data. Diffusion pseudotime plots made from deep learning features with accompanying ground truth class information. The first row has plots of the first two diffusion coordinates and the next row has plots of the second and third diffusion coordinates. Note: the model used to generate these plots is slightly different than the final one run in the paper. Figure S5. Visualization of learned features and single-cell data. t-SNE plot made from deep learning [file 12859_2020_3635_MOESM1_ESM.docx]

Supplemental Material


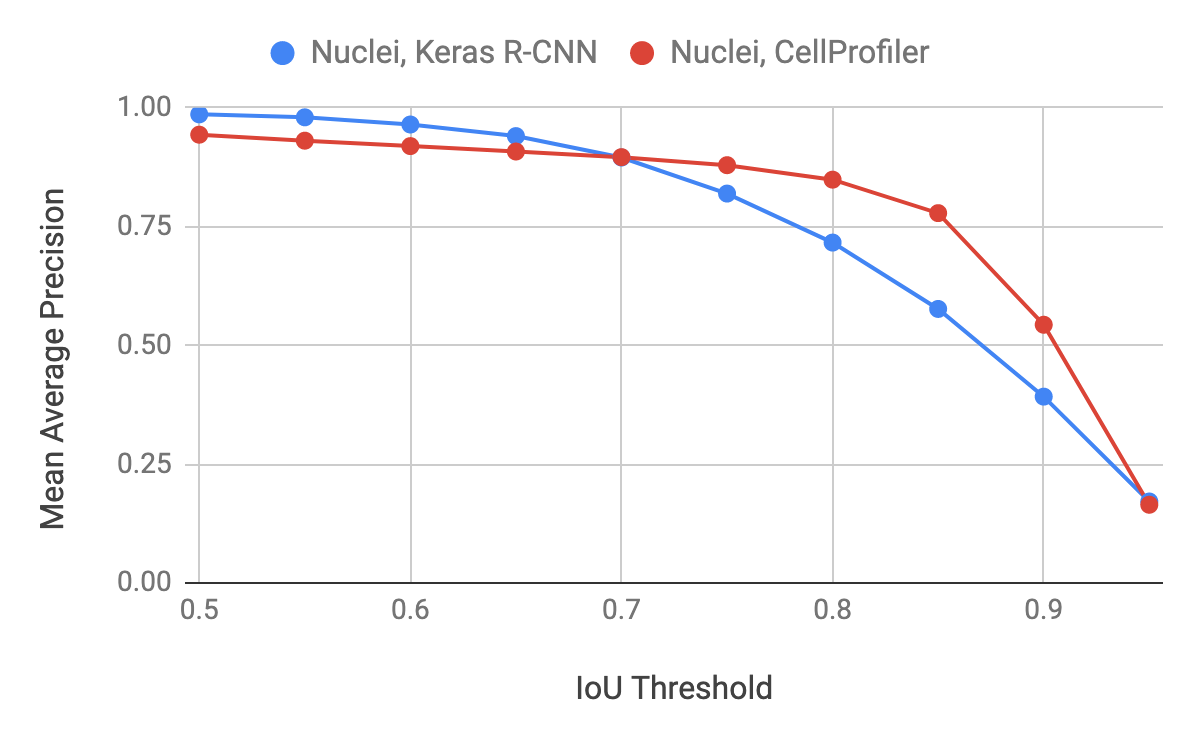

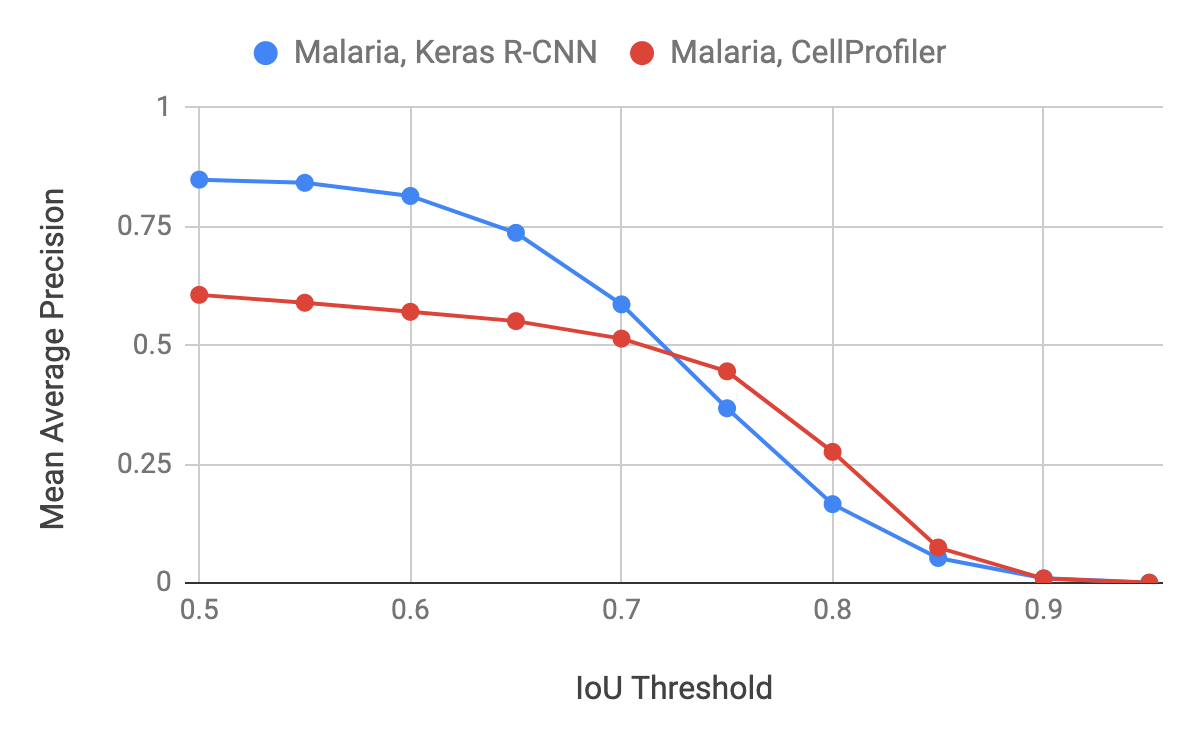


***Figure S1: Comparison of mean average precision curves for different IoU thresholds for Keras R-CNN versus CellProfiler on the nuclei and malaria datasets.*** *For nuclei, the mean average precision is 0.99 at a threshold of 0.5 for Keras R-CNN. For malaria, the mean average precision is 0.78 at a threshold of 0.5 for Keras R-CNN.*

*
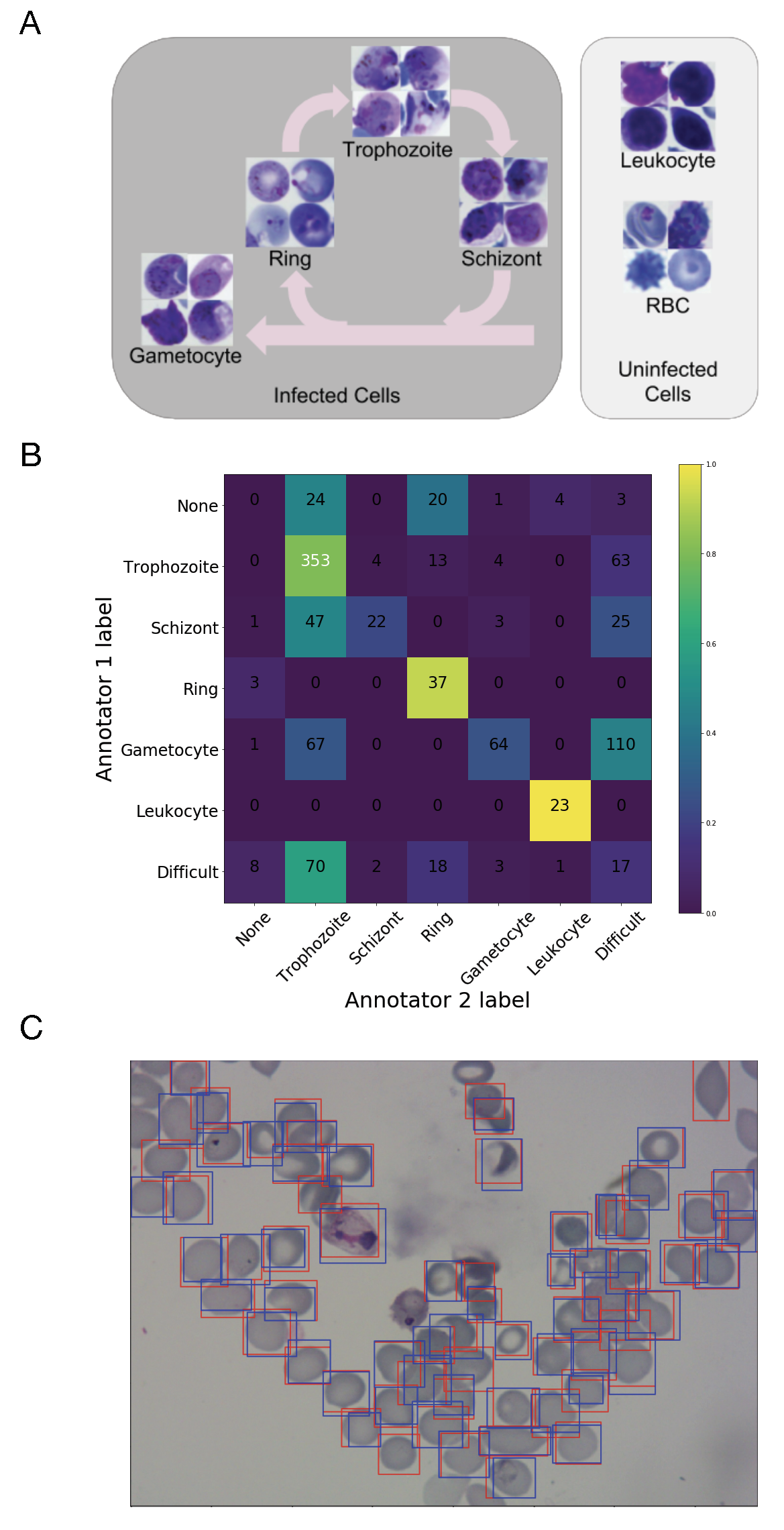
*

***Figure S2: Overview of* P. vivax *data and results.*** *The samples contain two classes of uninfected cells (red blood cells and leukocytes) and four classes of infected cells (gametocytes, rings, trophozoites, and schizonts) and have a heavy imbalance: more than 95% of all cells are uninfected, roughly the distribution in patient blood. A. Depiction of all relevant cell types found in human blood, including two types of uninfected cells and 4 types of infected cells in the* P. vivax *life cycle. The cycle on the left shows asexual development. Gametocytes come from sexual development and lead to transmission. B. Confusion matrix comparing annotations of two experts (colors normalized so that rows sum to 1); the significant signal off-diagonal speaks to the challenge for experts to agree upon the proper stage label for each cell. Experts were asked to identify relevant cells and label them as one of the cell types or difficult. C. Example of malaria-infected blood smear results. Red boxes are ground truth; blue boxes are predictions produced by Keras R-CNN.*

## Datasets

| Dataset | Organization | People |
| --- | --- | --- |
| Ex vivo samples from Brazil | Dept of Genetics, Evolution, Microbiology and Immunology. University of Campinas. Campinas-SP. Brazil.; Fundação de Medicina Tropical Dr. Heitor Vieira Dourado, Gerência de Malária, Manaus, Amazonas; Instituto Leônidas e Maria Deane, Fundação Oswaldo Cruz (FIOCRUZ), Manaus, Amazonas | Deepali Ravel, Stefanie Lopes, Marcus Lacerda, Fabio Costa |
| Ex vivo samples from Southeast Asia | Shoklo Malaria Research Unit of Professor François Nosten in North West of Thailand, Singapore immunology Network (SIgN); A*STAR | Benoit Malleret, Laurent Rénia, Francois Nosten |
| In vitro time course samples | Harvard School of Public Health | Gabriel Rangel, Odailton Amaral Nery, Marcelo U. Ferreira, Manoj Duraisingh |

***Table S1: Malaria datasets***

With this paper, we publicly release the malaria datasets in the hopes that it will bring more interest from computer scientists in improving the particular challenges that biological images present. Cells in brightfield microscopy images present several challenges. Like natural images, microscopy images have variations; for microscopy, these include illumination from the microscope, in cell shape, density, and color from variations in sample preparation, and have objects of uncertain class- even for experts. Unlike natural images, it is difficult to annotate the data with the expertise necessary for accurate training because of the lack of experts. There is also an inherent class distribution imbalance due to the dominance of uninfected red blood cells (RBCs).

This study used three sets of images consisting of 1364 images (~80,000 cells) with different researchers having prepared each one: from Brazil (Stefanie Lopes), from Southeast Asia (Benoit Malleret), and time course (Gabriel Rangel) (see Table S1). The data consists of two classes of uninfected cells (RBCs, leukocytes) and four classes of infected cells (gametocytes, rings, trophozoites, schizonts). All non-RBC objects were annotated by Deepali Ravel in Matthias Marti’s lab at Harvard School of Public Health and Stefanie Lopes in Fabio Costa’s lab in the Dr. Heitor Vieira Dourado Tropical Medicine Foundation. There were 6 labels used to cover possible cell types of interest: RBC, leukocyte, gametocyte, ring, trophozoite, and schizont. RBCs and leukocytes (white blood cells) are normally found in blood. An additional label, difficult, was used by annotators to mark cells that were not clearly in one of the cell classes - such cells were ignored in training. Annotators were permitted to mark some cells as difficult if not clearly in one of the cell classes; these difficult cases (~1% of the total and ~23% of the infected cells) are ignored in training. The data had a heavy imbalance: uninfected RBCs were more than 95% of all cells (versus uninfected leukocytes and infected cells).

## Keras R-CNN Dataset Schema

External data can be added if it is in the proper form. The following describes our schema, made up of a list of dictionaries corresponding to images.

- For each image, add a dictionary with keys *image*, *objects*
  - *image* is a dictionary, which contains keys *checksum*, *pathname*, *shape*
    - *checksum* is the md5 checksum of the image
    - *pathname* is the pathname of the image, put in full pathname
    - *shape* is a dictionary with keys *r*, *c*, *channels*
      - *c*: number of columns
      - *r*: number of rows
      - *channels*: number of channels
  - *objects* is a list of dictionaries, where each dictionary has keys *bounding_box, category*
    - *bounding_box* is a dictionary with keys *minimum*, *maximum*
      - *minimum*: dictionary with keys *r, c*
        - *r*: smallest bounding box row
        - *c*: smallest bounding box column
      - *maximum*: dictionary with keys *r*, *c*
        - *r*: largest bounding box row
        - *c*: largest bounding box column
    - *category* is a string denoting the class name

## Model

Deep neural networks are organized in interconnected layers of nodes. The layers in between the input and output, called hidden layers, try to learn the complex function which maps pixels to object labels. As input, nodes take the output [
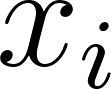
](http://www.codecogs.com/eqnedit.php?latex=%20x_i%20) for each node [
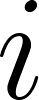
](http://www.codecogs.com/eqnedit.php?latex=i) from the layer below and add them in a weighted sum with bias [
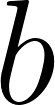
](http://www.codecogs.com/eqnedit.php?latex=b): [
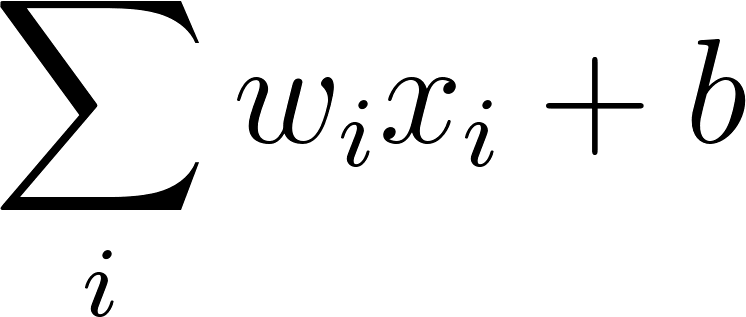
](http://www.codecogs.com/eqnedit.php?latex=%20%5Csum_i%20w_ix_i%20&plus;%20b%20). A node’s output is the result of applying a nonlinear activation function to the input, usually the rectified linear activation function (ReLu).

Convolutional Neural Networks (CNNs) have gained the most popularity because fewer parameters means easier training and improved generalizability[[22–25]](https://paperpile.com/c/pSMC5I/tzNbQ+5G9rp+0JEw7+Y6Jab). Nodes in CNNs are only connected to a subset of nodes from the previous layer. CNNs consist of alternating convolutional and pooling layers: convolutional layers apply multiple filters to extract features from local regions of their input while pooling layers help control overfitting by reducing the number of parameters.

Object detection networks like Faster R-CNN[[6]](https://paperpile.com/c/pSMC5I/mWvz3) can locate objects of interest without segmentation. Faster R-CNN consists of 2 modules: the first uses a region proposal network (RPN) that proposes object regions and the second is a Fast R-CNN object detector that classifies the region proposals[[9]](https://paperpile.com/c/pSMC5I/RY3EX). To save time, RPN and Fast R-CNN share a convolutional network. RPNs take an image and output rectangular object proposals with associated objectness probability estimates. The full image is processed through the shared convolutional network to get a feature map; a fully connected network is slid over square regions of the feature map, each time yielding a vector; the vector is fed into 2 branches of fully connected layers, one for box classification (object class vs. background) and one for bounding box regression, in which the corner coordinates of boxes are adjusted relative to anchor boxes of predetermined size and shape. The bounding boxes and their associated object scores are fed into the Fast R-CNN module as region proposals.

Fast R-CNN then takes the feature map and object proposals, and outputs per-class coordinate offsets and per-class probability estimates. Each proposal is projected onto the feature map and sent to the Region of Interest pooling layer, which uses max pooling to convert the features inside any valid region of interest into a small fixed sized feature map. The small feature map is run through fully connected layers to become a feature vector; the feature vector is fed into 2 branches of fully connected layers, one for softmax classification to classify the final detections and one for bounding box regression to get the coordinates of the final detections. Finally, redundant detections are removed by non-maximum suppression, which removes detections with intersection over union (IoU) above a set threshold relative to a detection with higher class score.

Like other machine learning algorithms, training is done by minimizing a loss function. Faster R-CNN combines four losses to make a multitask loss. Each module has a classification and regression loss associated with it. Both RPN and RCNN’s classification losses are categorical cross-entropies; in the case of RPN, there are just two classes- objects vs. background- so it is a binary cross-entropy, while for RCNN, the cross-entropy is over all classes. The regression losses for RPN and RCNN are a smooth L1 loss. The total loss is a mean over all detections with each detection’s loss calculated as

[
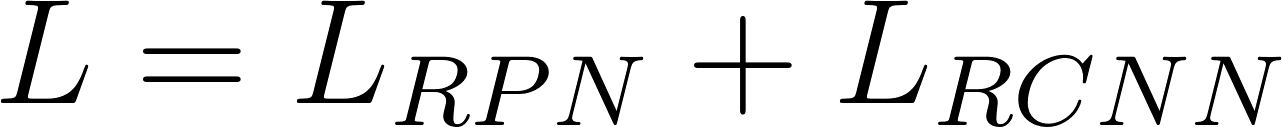
](https://www.codecogs.com/eqnedit.php?latex=%20L%20%3D%20L_%7BRPN%7D%20%2B%20L_%7BRCNN%7D%250)

[
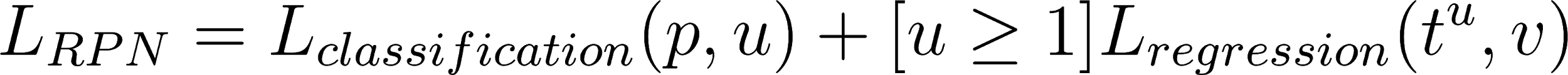
](https://www.codecogs.com/eqnedit.php?latex=L_%7BRPN%7D%20%3D%20L_%7Bclassification%7D%20(p%2C%20u)%20%2B%20%5Bu%20%5Cgeq%201%5D%20L_%7Bregression%7D(t%5Eu%2C%20v)%20%250)

[
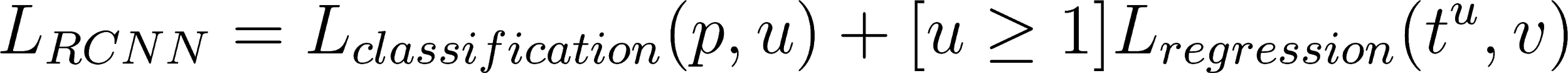
](https://www.codecogs.com/eqnedit.php?latex=L_%7BRCNN%7D%20%3D%20L_%7Bclassification%7D%20(p%2C%20u)%20%2B%20%5Bu%20%5Cgeq%201%5D%20L_%7Bregression%7D(t%5Eu%2C%20v)%20%250)

[
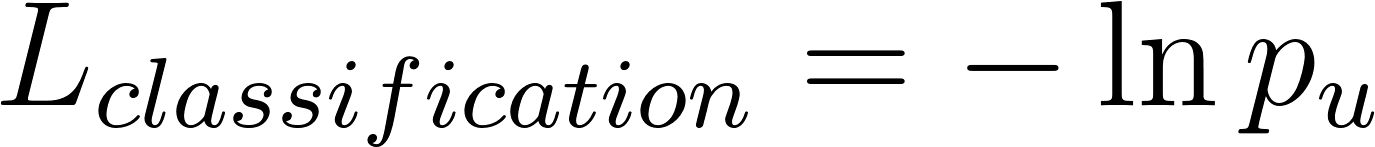
](https://www.codecogs.com/eqnedit.php?latex=L_%7Bclassification%7D%20%3D%20-%5Cln%20p_u%20%250)

[
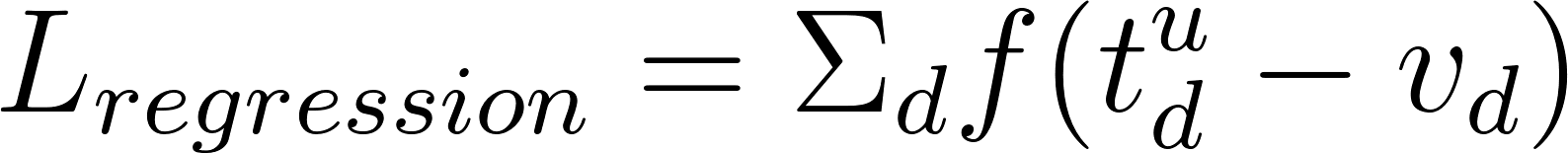
](https://www.codecogs.com/eqnedit.php?latex=L_%7Bregression%7D%20%3D%20%20%5CSigma_d%7D%20f(t_d%5Eu%20-%20v_d)%250)

[
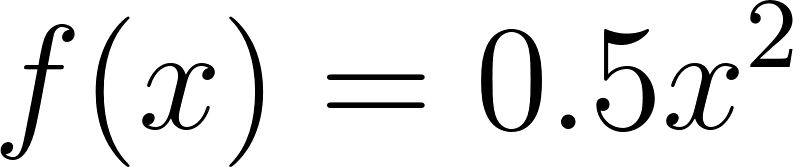
](https://www.codecogs.com/eqnedit.php?latex=f(x)%20%3D%200.5x%5E2%250) if [
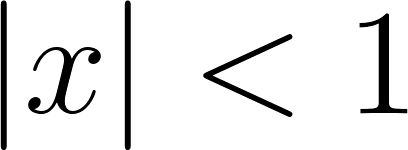
](https://www.codecogs.com/eqnedit.php?latex=%5Cleft%7C%20x%20%5Cright%7C%20%3C%201%250), else [
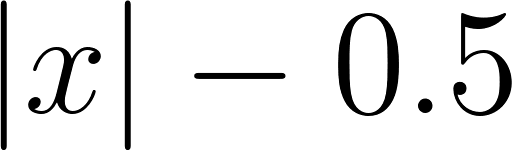
](https://www.codecogs.com/eqnedit.php?latex=%5Cleft%7C%20x%20%5Cright%7C%20-%200.5%250)

where [
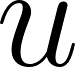
](https://www.codecogs.com/eqnedit.php?latex=u%250) is the ground truth class (0 for the background class and >0 for the object classes), the Iverson indicator bracket function indicates whether the ground truth class is background or not, [
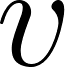
](https://www.codecogs.com/eqnedit.php?latex=v%250) is the ground truth regression target offsets, [
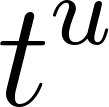
](https://www.codecogs.com/eqnedit.php?latex=t%5Eu%250) is the predicted regression offsets for class [
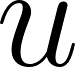
](https://www.codecogs.com/eqnedit.php?latex=u%250), [
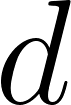
](https://www.codecogs.com/eqnedit.php?latex=d%250) is looped over coordinates, and [
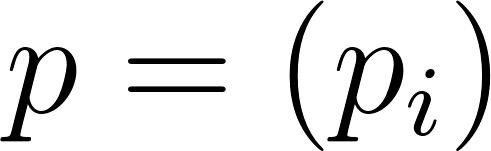
](https://www.codecogs.com/eqnedit.php?latex=p%20%3D%20(p_i)%250) is the probability distribution over all classes. Note that in the context of the RPN, there are two classes: object and background; in the context of RCNN, there is a background class as well as N object classes. There are also two sets of predicted regression offsets, ground truth target offsets, and predicted probability distributions for the RPN and RCNN.

## Preprocessing

An important preprocessing step to try to get colors to match better across image sets was to rescale each channel of an image between the minimum and maximum intensities (from the corresponding channel). Images are equalized with contrast limited adapted histogram equalization.

All experiments were performed with training 512 by 512 crops. Every epoch has a new random cropping, and bounding boxes are clipped to the 512 by 512 crop. As is, the training set is highly imbalanced towards RBC, so to create a more balanced training set, we augmented crops containing underrepresented object types and limited the number of crops containing only RBCs to be around 5% of the total number of crops. Classes were considered underrepresented if they weren’t RBC and weren’t the 2nd most frequent class if the 2nd most frequent class had more than 4 times the number of examples as the 3rd most frequent class. To further enhance the training set, all crops were vertically and horizontally flipped and bounding boxes were shifted a random number of pixels between 2 and 8.

## Training

Training and validation were split 50/50 with a simple random sampling. Validation is used to tune the learning rate and the anchor box size and shape hyperparameters. We varied the learning rate between 0.1 and 0.00001 to determine the most effective order of magnitude. The anchor box hyperparameters should represent prior knowledge of the size and shape of the objects of interest; each object of interest should be expected to fit within at least one of the anchor boxes. We tried a few variations of these hyperparameters but found the results were robust to small changes in these values. Our optimizer was Adam with learning rate 0.001 for both datasets. DSB was trained for 700 epochs while Malaria was trained for 300 epochs.

## P. Falciparum Results


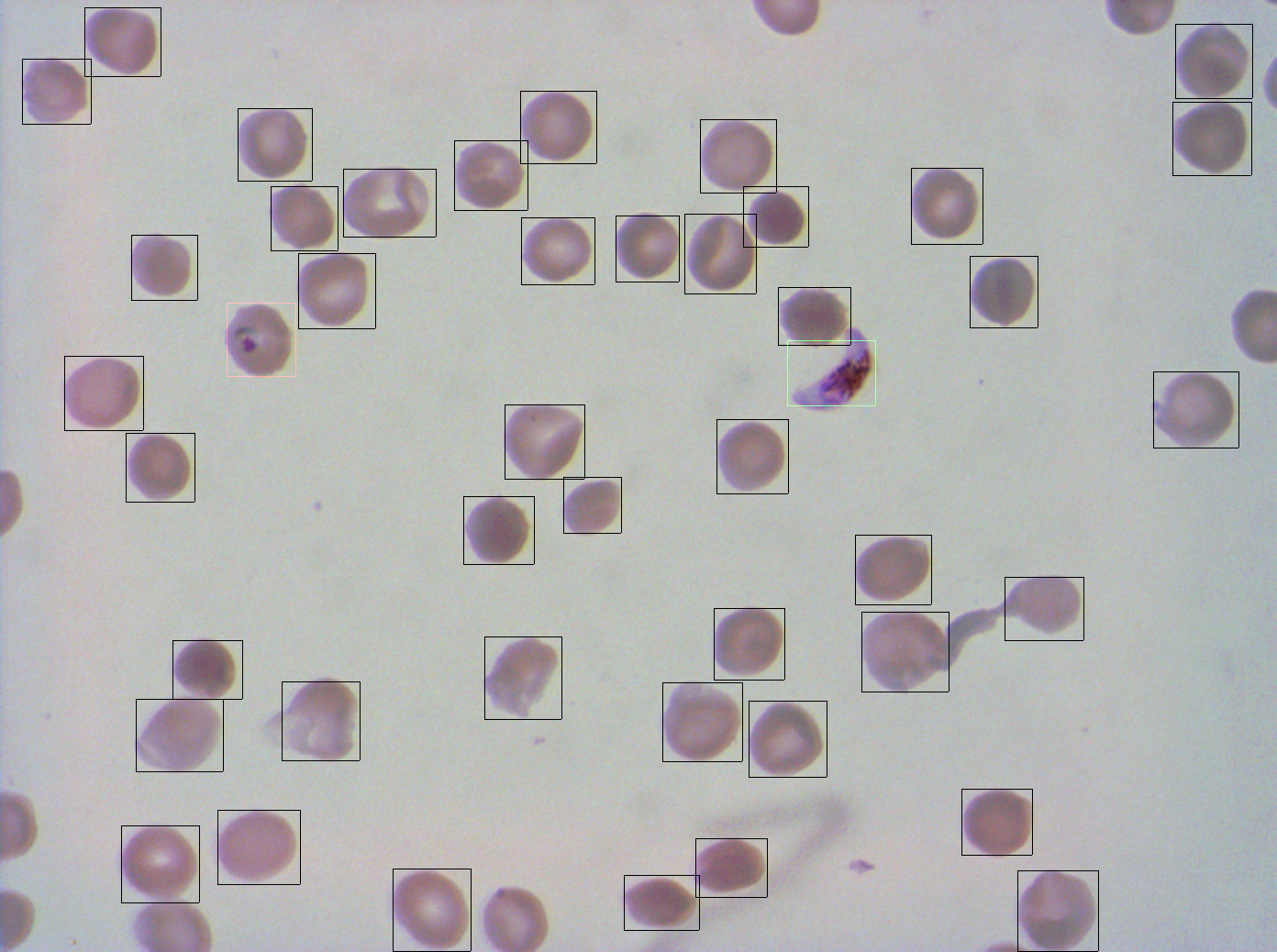


| **Average Precision** | | |
| --- | --- | --- |
|  | Deep Learning | Traditional |
| rbc | 0.91 | 0.91 |
| ring | 0.83 | 0.58 |
| gametocyte | 0.91 | 0.81 |

***Figure S3. Results for* P. falciparum**

We carried out some preliminary work using one slide from an *in vitro* culture of *P. falciparum* containing mature gametocytes and asexual rings with purified RBCs. The slide was prepared by the Marti lab with 350 images at 100X in oil immersion.

We compared the results from the traditional approach using Random Forest with 1000 trees and class reweighting to help reduce class imbalance effects on CellProfiler features extracted from cells identified in the CellProfiler segmentation pipeline to using Faster R-CNN. This resulted in an average precision of the RBC class of the model being comparable to the traditional approach because some cells were not detected by Faster R-CNN, but the gametocyte stage average precision was 10 percentage points higher for deep learning. Better performance for gametocytes was mostly from Faster R-CNN’s ability to detect objects without oversegmentation problems. The promising results found for *P. falciparum* prompted us to implement Faster R-CNN for biological applications in particular.

## Evaluation

For nucleus detection, we used training images from a subset of the 2018 Data Science Bowl (DSB) dataset *BBBC038* and testing images from a subset of the human U2OS cell dataset *BBBC022*[[14,15]](https://paperpile.com/c/pSMC5I/skGGs+SeKa5). Malaria was evaluated against a holdout set from a different experiment from a different lab with a different microscope system than training.

Comparisons were done with different threshold values using mean average precision across labs to balance localization and classification accuracy (Supplementary Figure S1). The mean average precision score is a standard metric for object classification tasks that combines per-class recall and precision of intersection over union (IoU) values between predicted boxes and ground truth. In short, MAP is a single metric that summarizes the precision of the predicted objects sorted by probability that correspond to ground truth objects.

In the case of object detection, true positives and false positives are determined by comparing the maximum IoU over all ground truth to a set threshold. IoU of two rectangles is their intersection divided by their total area. Average precision is the area under the precision-recall curve and varies between 0 and 1.

[
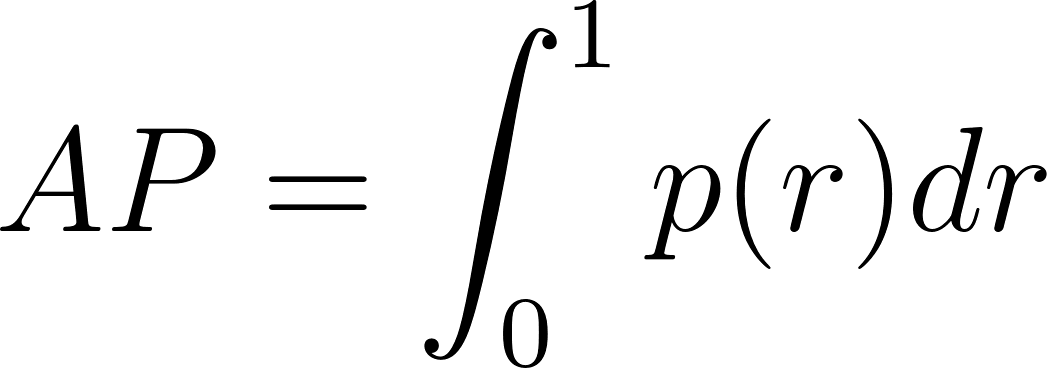
](http://www.codecogs.com/eqnedit.php?latex=AP%20=%20%5Cint_0%5E1%20p(r)dr)

Equation 1. Definition of average precision, where [
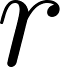
](http://www.codecogs.com/eqnedit.php?latex=r) is recall and [
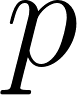
](http://www.codecogs.com/eqnedit.php?latex=p) is precision as a function of recall. [
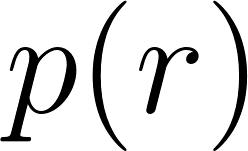
](http://www.codecogs.com/eqnedit.php?latex=p(r)) is the precision measured at recall [
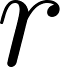
](http://www.codecogs.com/eqnedit.php?latex=r).

The CellProfiler comparisons used CellProfiler 3.0 (<https://github.com/CellProfiler/CellProfiler>). For nucleus cell detection, bounding boxes around the CellProfiler generated segmentations were used in our evaluation.

## Feature Analysis


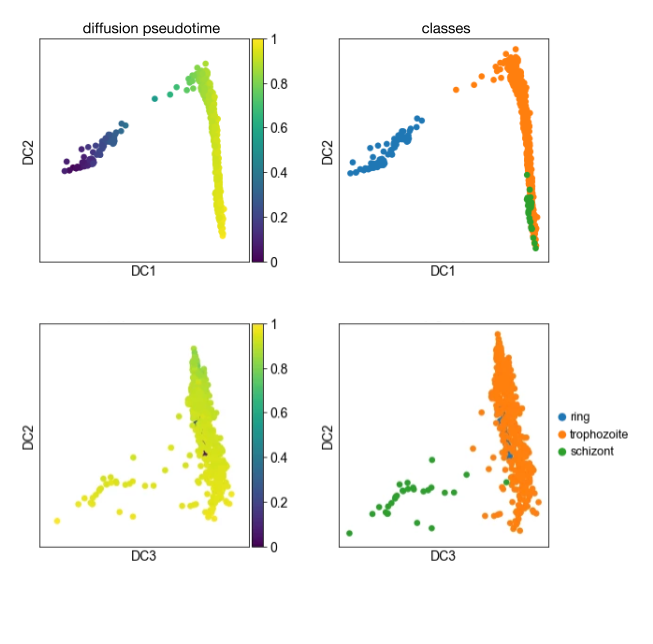


***Figure S4. Visualization of learned features and single-cell data.*** *Diffusion pseudotime plots made from deep learning features with accompanying ground truth class information. The first row has plots of the first two diffusion coordinates and the next row has plots of the second and third diffusion coordinates. Note: the model used to generate these plots is slightly different than the final one run in the paper.*


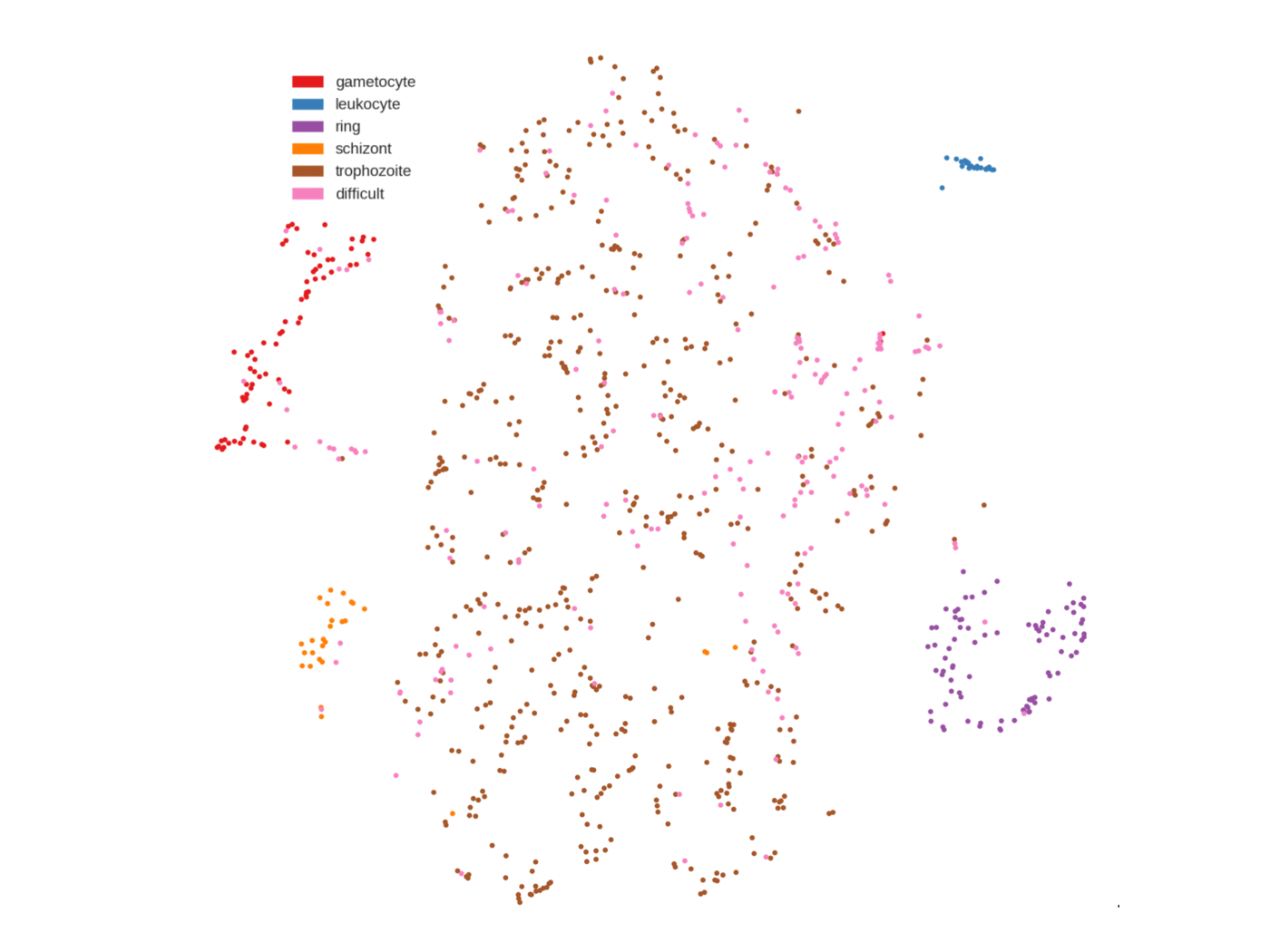


***Figure S5. Visualization of learned features and single-cell data.*** *t-SNE plot made from deep learning features colored by ground truth class information. Note: the model used to generate these plots is slightly different than the final one run in the paper.*

Aside from performing classification to match human experts, deep learning has the potential to also provide insights into biological processes. We therefore also developed scripts to perform certain analyses for the malaria application, given that the stages of parasite development are continuous and experts often have varying assessments. A method called diffusion pseudotime[[19]](https://paperpile.com/c/pSMC5I/bfAFw) can be used on diffusion maps to find temporal order in the data based on a set of features by calculating random walk based distances from diffusion map Euclidean distances starting with a root cell. The approach is effective in cases where highly similarity between cells indicates a similar position along a chronological continuum.

Supplementary Figure S4 shows the results of applying diffusion pseudotime to the features of asexual stages of malaria learned by the deep learning image classification model. We use only the asexual stages because they have a known progression. Temporal ordering, as determined by the diffusion algorithm itself, is shown on the left, and ground truth class labels are shown on the right. The top row of charts in the figure shows the first two diffusion coordinates, which are the first eigenvectors, and show the main progression of the cells. Having chosen a random ring cell as the root, diffusion pseudotime was able to identify other rings and order trophozoites as the next stage, followed by schizonts. The relative closeness of trophozoites and schizonts compared to rings and other stages indicates that rings are more distinct than the others. The second row shows the second and third diffusion coordinates and the secondary progression, reflecting the final steps in the progression into the schizont cluster. The diffusion pseudotime ordering of the cells matches the cell progression that we expect, which suggests the learned features capture important and biologically meaningful underlying cell information.

Supplementary Figure S5 shows the results of another unsupervised feature reduction technique, T-distributed stochastic neighborhood embedding (t-SNE). The plot shows that the different stages of development, including sexual gametocytes and non-infected leukocytes, can be grouped together just using the model’s learned features. There is also a relatively large expanse of trophozoites and difficult cells, which confirms the observation that the trophozoite stage is the most diverse in appearance and hard to clearly stage. Together with Supplementary Figure S4, this is indicative of the power of the model’s learned features.

## Object Detection Time Comparison

To compare the inference times of different object detection methods, we refer to Lin et al. [26]. Faster R-CNN has one of the best performances second only to RetinaNet 101-800 but is faster.


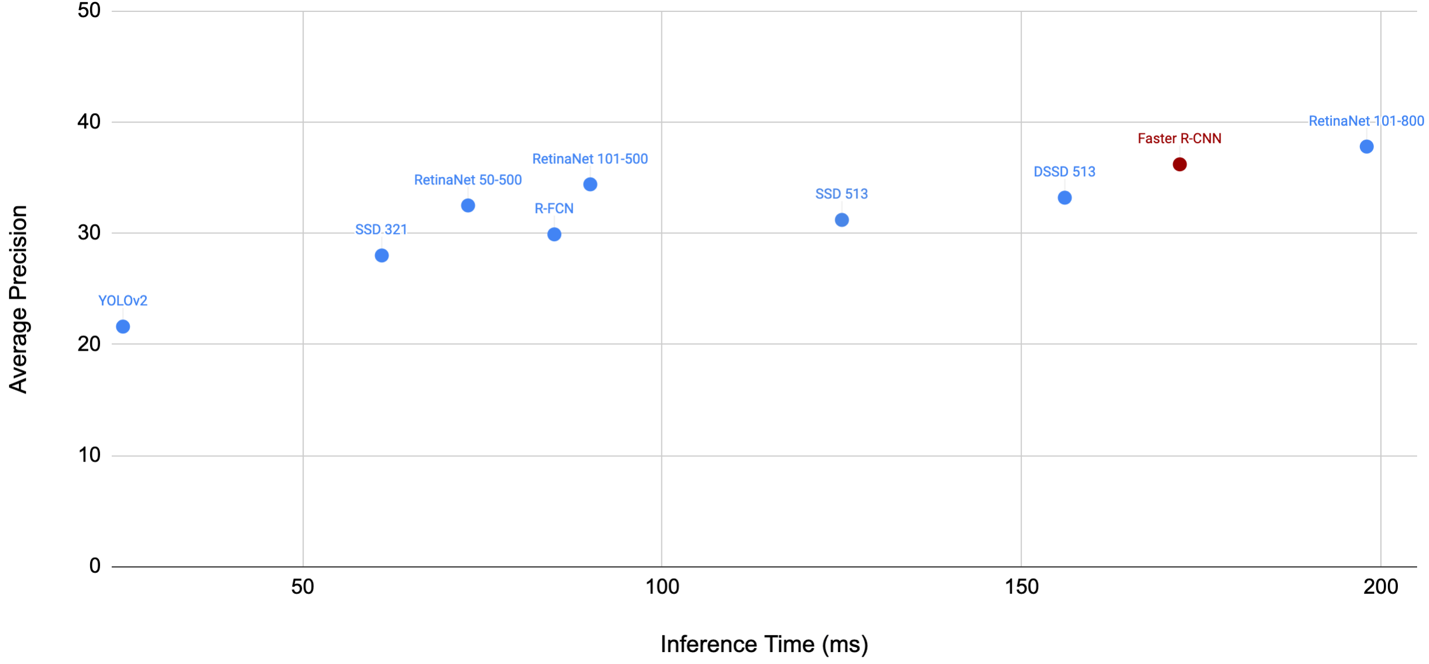


***Figure S6. Inference time comparison across common object detection methods.***
